# Supplementary material for: Leader extraversion and team performance: A moderated mediation model
Source: PLoS One. 2022 Dec 9;17(12):e0278769. doi: 10.1371/journal.pone.0278769 (PMC9733865; doi:10.1371/journal.pone.0278769)
Supplement: Attachment — Submitted filename: PONE-D-22-19951.docx [file pone.0278769.s002.docx]

*Thank you for inviting me to review a manuscript entitled “Leader Extraversion and Team Performance: A Moderated Mediation Model.”*

Although the paper is well written but requires several changes, a few of them are as follows

**Abstract**

Add a background sentence before stating the objective of the study. In addition, when and how data was collected [I mean how respondents were selected; in which month or year data was collected]. Besides, also highlight which software has been used for the analysis. Finally, add a conclusion sentence and add some implications.

**Introduction**

This section was very limited. There is a need to add more content [why this research is needed] and highlight the research gap by covering each relationship proposed in the model. Based on the research gap, draw the research questions and objective and add the contribution paragraph in the last. I will suggest you cite recent work instead of old work. I will suggest moving to figure 1 in the next section's theoretical background after the hypotheses end.

Theoretical background and hypotheses

I could not find any explanation for the distal-proximal motivational and situational strength theories. There is a need to define the assumptions of both theories first. After that, based on theoretical argument, construct your hypotheses. Referring to line 68 [author argues no work]. However, several studies investigate the mediation of work engagement.

Tanskanen, J., Mäkelä, L. & Viitala, R. Linking Managerial Coaching and Leader–Member Exchange on Work Engagement and Performance. *J Happiness Stud* **20**, 1217–1240 (2019). <https://doi.org/10.1007/s10902-018-9996-9>

Gutermann, D., Lehmann-Willenbrock, N., Boer, D., Born, M. and Voelpel, S.C. (2017), How Leaders Affect Followers’ Work Engagement and Performance: Integrating Leader−Member Exchange and Crossover Theory. Brit J Manage, 28: 299-314. <https://doi.org/10.1111/1467-8551.12214>

**Methodology**

Add a sampling section and explain further how respondents have been selected. In addition, add each constructs item used in the study.

**Results**

Divide the results section into the measurement model and structural model. In particular, in the measurement model, use EFA to better look into the multicollinearity issues; the current results are insufficient to comment further.

**Discussion**

This section needs substantial changes. Initially, divide this section into three sub-parts: theoretical and practical implications, limitations, and future research. Secondly, discuss your result in the context of prior studies which is missing. Theoretical and practical implications require to be supported by citations.
